# Biocatalytic Membranes for Carbon Capture and Utilization

**DOI:** 10.3390/membranes13040367

**Published:** 2023-03-23

**Authors:** Jialong Shen, Sonja Salmon

**Affiliations:** Department of Textile Engineering, Chemistry and Science, Wilson College of Textiles, North Carolina State University, Raleigh, NC 27695-8301, USA; jshen3@ncsu.edu

**Keywords:** biocatalyst, carbonic anhydrase, CO_2_ capture, CO_2_ reduction, enzyme, formate dehydrogenase, immobilization, membrane

## Abstract

Innovative carbon capture technologies that capture CO_2_ from large point sources and directly from air are urgently needed to combat the climate crisis. Likewise, corresponding technologies are needed to convert this captured CO_2_ into valuable chemical feedstocks and products that replace current fossil-based materials to close the loop in creating viable pathways for a renewable economy. Biocatalytic membranes that combine high reaction rates and enzyme selectivity with modularity, scalability, and membrane compactness show promise for both CO_2_ capture and utilization. This review presents a systematic examination of technologies under development for CO_2_ capture and utilization that employ both enzymes and membranes. CO_2_ capture membranes are categorized by their mode of action as CO_2_ separation membranes, including mixed matrix membranes (MMM) and liquid membranes (LM), or as CO_2_ gas–liquid membrane contactors (GLMC). Because they selectively catalyze molecular reactions involving CO_2_, the two main classes of enzymes used for enhancing membrane function are carbonic anhydrase (CA) and formate dehydrogenase (FDH). Small organic molecules designed to mimic CA enzyme active sites are also being developed. CO_2_ conversion membranes are described according to membrane functionality, the location of enzymes relative to the membrane, which includes different immobilization strategies, and regeneration methods for cofactors. Parameters crucial for the performance of these hybrid systems are discussed with tabulated examples. Progress and challenges are discussed, and perspectives on future research directions are provided.

## 1. Introduction

Carbon dioxide (CO_2_) interacts with atmospheric, oceanic, and terrestrial biospheres [1] and is a part of feedback mechanisms that are responsible for natural glacial cycles [2]. Within the past century, anthropogenic CO_2_ emissions have skyrocketed, causing an abrupt increase in atmospheric CO_2_ levels [3] which is speeding up global warming and risks irreversible climate changes [4]. Carbon capture technologies that reduce the emissions from large point sources and capture legacy emissions directly from air are necessary to combat this crisis [5]. Converting captured CO_2_ into valuable chemical feedstocks and products that replace current fossil-based materials is increasingly important for its double benefit and has incentivized the growth of an emerging market for carbon utilization [6].

Already, there are four types of industrially used CO_2_ capture technologies. They include absorption in chemical or physical solvents, adsorption on solid absorbents, cryogenic processes, and membrane-based separations [7]. Each have their own advantages and limitations depending on the specific implementation conditions, such as temperature, pressure, and CO_2_ concentrations. It is therefore common to combine more than one technology in efforts to reduce the overall cost of the capture process. For example, a conventional membrane separation process can be assisted by a cryogenic unit to improve CO_2_ purity to a level that is not attainable by the membrane alone [8]. In another hybrid example, the porous structure of a gas–liquid membrane contactor (GLMC) is used to increase the contact surface areas of a gas stream with conventional chemical absorption solvents to achieve higher CO_2_ capture efficiency with a smaller footprint [9]. The development of more sophisticated hybrid systems that combine the relatively newer concepts of metal organic frameworks (MOFs), ionic liquid (IL), and enzyme-based CO_2_ capture and reactor systems, together with conventional CO_2_ capture technologies, offer new possibilities for carbon capture and utilization applications [10]. By comparing their features, this review examines the range of hybrid technologies that employ both enzymes and membranes, and provides critical analyses of the promises and challenges within the field of biocatalytic membranes for carbon capture and utilization (CCU). This review provides a concise account of both membrane and biocatalysis concepts relevant for CCU. The analyses and discussions are intended to stimulate inspiration for novel ideas, and especially, to encourage collaboration among researchers from different academic backgrounds.

### 1.1. Enzymes for CO_2_ Capture and Utilization

#### 1.1.1. Carbonic Anhydrases

Among the many chemical reactions catalyzed by enzymes in biological systems, the hydration and dehydration reactions of CO_2_ and bicarbonate (Equation (1)) related to cell respiration and blood pH homeostasis are among the most critical. Carbonic anhydrases (CAs) are a ubiquitous class of metal-containing enzymes found in all domains of life. At ideal conditions, CAs can catalyze the conversion of up to the order of one million molecules of substrate per molecule of enzyme per second [11]. Interest in using CA for engineering purposes was initially limited to CO_2_ separation from life supporting closed spaces [12], such as submarines and spacecraft, or in biomedical devices, such as artificial lungs [13]. Now interest has been rekindled for its potential industrial-scale use [14] in mitigating the negative effects of CO_2_ on climate change.

CO_2_ + H_2_O ↔ H^+^ + HCO_3_^−^ (1)

In recent years, CA has been evaluated both in a dissolved form [15] or immobilized on packing materials [16,17] as a promoter in conventional CO_2_ chemical absorption processes for enhancing CO_2_ capture from power plant emissions using benign low regeneration energy solvents, such as aqueous solvents containing potassium carbonate, K_2_CO_3_. These inorganic salt-based solvents enhanced by CA offer the special advantage of minimizing pore wetting for novel gas–liquid membrane contactor applications, which is discussed in detail below. With the goal of finding lower cost synthetic alternatives, various CA mimic enzymes such as Zinc-based [18] and Cobalt-based [19] catalysts have been devised. Because water (moisture) is essential for CA enzymes to provide an enhancement effect, membrane developments that utilize CA and its mimics focus on “wet” membranes as the catalytic reaction cannot occur in dry membranes [20].

Like many other enzymes, CAs generally have low tolerance for elevated temperature environments, such as those encountered in the CO_2_ stripper of reactive absorption processes. However, some CAs are naturally thermostable [14], and non-natural variants have been made by protein engineering to create ultra-thermostable CAs that can tolerate temperatures of up to 107 °C with pH > 10.0 in amine solvents [21]. Furthermore, enzyme immobilization can circumvent CA instability by retaining CAs in the lower temperature absorber column, thus preventing them from being exposed to the high temperature environment, and can also stabilize CAs against denaturing in harsh solvents for improved longevity [22].

#### 1.1.2. Formate Dehydrogenases

Due to its vast quantity, captured CO_2_ needs to be stored permanently, or ideally turned into valuable materials that displace CO_2_ emissions from traditional processes. Such processes include the cement and chemical industries, which account for 7% of global CO_2_ emissions and 7% of all oil extracted, respectively [23]. Formate dehydrogenase (FDH) catalyzes the reversible reduction of CO_2_ to formic acid (Equation (2)), a commodity chemical with an existing market of more than one million tons/year [24]. Theoretically, the market size would grow much larger if efforts to use formate for liquid hydrogen fuel storage [25] or as a carbon source for microbial growth and biosynthesis of higher carbon chemicals [26,27] can be realized.

CO_2_ + H^+^ + 2e^−^ ↔ HCOO^−^ (2)

The two major categories of FDH are metal-independent and metal-dependent. Metal-independent FDHs have limited CO_2_ reduction activity [28], preferring to catalyze the formate oxidation reaction. CO_2_ reduction requires a proton and two reducing electrons, which are supplied by enzyme cofactors, such as the reduced form of nicotinamide adenine dinucleotide (NADH). To convert each mole of CO_2_, an equal molar amount of NADH is converted to the oxidized NAD^+^ form. This means that NADH must be continuously supplied or regenerated to operate enzymatic membrane reactors continuously. Efficient regeneration of natural cofactor or artificial electron donors and carriers is, therefore, the bottleneck in enzyme efficiency that has spurred many innovative strategies reflected in the recent literature in this field. For example, Song et al. [29] covalently linked copper nanoparticles (CuNPs) with FDH for the regeneration of the enzyme cofactor NADH that is also tethered on the FDH through a flexible polyethylene glycol (PEG) swing arm. In addition, the rhodium bipyridine complex has been widely used to modify electrode surfaces for cofactor regeneration [30].

#### 1.1.3. Enzyme Cascade with Other Oxidoreductases

Enzymatically produced formic acid has many downstream pathways for utilization. Enzymatic conversion to formaldehyde and methanol, both of which are among the top 10 petrochemicals produced in the world, is of interest in the search for sustainable low-temperature processes [28]. Redox reactions of formic acid catalyzed by formaldehyde dehydrogenase (FaldDH) and then alcohol dehydrogenase (ADH) produce formaldehyde and methanol in sequence. Such an enzyme cascade reaction that incorporates relevant enzymes in the same system in close proximity to each other could have additive rate enhancement effects due to raised local substrate concentration and reduced product inhibition [28]. However, there are several common limitations on current oxidoreductase for CO_2_ reduction systems that need to be addressed. These include low enzyme activity in the carbon reduction direction, low efficiency of cofactor regeneration, low efficiency of electron transfer in cofactor-free systems, and CO_2_ solubility and mass transfer limitations [31]. Research activities in protein engineering, enzyme immobilization [32], and reactor design and integration with complementary systems [33] have all contributed to overcoming these limitations [31]. Details of recent advances in performance and understanding of cascade systems involving membranes are discussed below.

#### 1.1.4. Enzyme Immobilization

Enzymes are commonly immobilized on solid carriers—membranes being one of the most versatile—to improve their stability, facilitate their reuse, and reduce overall processing and chemical conversion costs [34]. Membranes, as enzyme carriers, can either be fabricated before (pre-existing) or during (formed in situ) the enzyme immobilization process.

On pre-existing (often commercially available) membrane carriers, enzymes are immobilized through various mechanisms including adsorption, covalent bonding, and affinity binding [32]. Sun et al. [35] used water plasma for treating a polyvinylidene fluoride (PVDF) flat sheet membrane, followed by silanization to introduce amine and epoxide groups on the membrane surface for subsequent covalent attachment of enzymes. Another versatile surface coating reagent, dopamine (DA), can impart amine functionalities on almost any type of material surface. Sun et al. co-deposited polyethylenimine (PEI) with DA on PVDF and polyethylene (PE) membranes and covalently attached CA enzyme through glutaraldehyde (GA) crosslinking with high activity recovery and excellent reusability for enhancing CO_2_ mineralization through CaCO_3_ precipitation [36]. In a technique that is especially suitable for membrane carriers, enzymes can be immobilized by direct and deliberate membrane fouling. Luo et al. [37] used a simple pressure driven filtration system to immobilize FDH, FaldDH, and ADH into flat sheet membrane pores for biocatalytic production of methanol from CO_2_ without the need for chemical reagents.

Enzymes can also be immobilized in matrices by encapsulation or entrapment mechanisms during the phase inversion process of membrane fabrication. Ren et al. [38] encapsulated CA in zeolitic imidazolate framework 8 (ZIF-8), a type of MOF, and embedded the biocatalytic MOF in a poly(vinyl alcohol)/chitosan (PVA/CS) composite membrane. In a CaCO_3_ precipitation test, the membrane structures formed during the immobilization process assisted biocatalyst recovery and reuse and exhibited a 1.63-fold improvement, compared with non-membrane MOF containing CA. Wen et al. [39] created CA nanoflowers, prepared by co-crystallization with Cu^2+^ and Zn^2+^ metal ions, and then embedded these in a CS/PVA hydrogel membrane. The amount of CaCO_3_ produced by the biocatalytic composite membrane was nine-fold and two-fold higher compared with the free CA or dispersed nanoflowers alone, respectively. Enzyme immobilization by entrapment is the method most commonly used to fabricate mixed matrix membranes (MMM) with CA, or with CA mimics, for facilitated CO_2_ transport separation applications [19].

While enzyme immobilization is its own extensive discipline, the introduction above should suffice for purposes of the current review topic. Interested readers are referred to classical protocol compilations for more detailed discussions about specific techniques for enzyme immobilization [40,41]. In addition, as awareness of biomimetic CO_2_ mitigation solutions increases, the field of CA immobilization has matured. These advances are found in recent review articles on CA immobilization for CO_2_ capture technologies [42], its industrial implementation [43], and its use in reactive absorption processes [44]. Reviews on enzyme immobilization for biocatalytic membranes in general are also available [32].

#### 1.1.5. Comparisons of Biocatalysts with Electrocatalysts for CO_2_ Reduction Reaction

When the reducing electrons are supplied by an external power source, the biocatalyst is effectively functioning as an electrocatalyst. However, there are several characteristics that make biocatalysts different in term of advantages and disadvantages. Enzymes have three-dimensional active sites that are able to have very specific interactions with a particular substrate from a mixture of similar compounds [45]. This high selectivity is one of the most sought-after features of biocatalysts. However, the drawback of high substrate selectivity is that the CO_2_ reduction reaction can only proceed one step at a time by matching each specific substrate with a specific type of enzyme. This means a cascade of multiple enzyme systems is needed for complete oxidation or reduction of substrate to product. An example of the intricate reaction cascades utilized by nature is the Krebs’s cycle, which is essential to the energy generation in cells [46]. Secondly, enzymes are complex folded long-chain protein molecules that are, for the most part, insulators against electron transfers. Therefore, effectively “wiring” enzymes to electrodes becomes especially challenging [47]. This is not the case for inorganic electrocatalysts used for CO_2_ reduction reactions, including metal alloys, metal oxides, metal chalcogenides and others, where increasing selectivity and current density and reducing over-potential, are the major research obstacles within the field [48]. Recently, Saxena et al. reported copper selenide [49] and cobalt telluride [50] that are able to reduce CO_2_ to C2 products, such as acetic acid, with greater than 80% Faradaic efficiency (FE) and 75% selectivity at a low applied potential. In a nickel selenide electrocatalyst system, the selectivity of the product can be controlled by the applied potential, and an FE of over 98% can be achieved for acetic acid at lower applied potential [51]. By fine tuning the CO intermediate adsorption energy on the active site using a bimetal copper cobalt selenide electrocatalyst, an FE of 100% towards C2 products such as ethanol and acetic acid was achieved [52]. For comparison, the FE of electrocatalysts used for CO_2_ reduction reactions in the literature vary significantly from 3% to over 90% [48], while in a limited number of reports, the FE of biocatalysts varied from 10% for a three enzyme cascade system that produced methanol by cofactor-free direct electron transfer [53] to 23% for a single immobilized enzyme and cofactor regenerating hybrid system that produced formic acid [29]. Both electrocatalytic and bio-electrocatalytic CO_2_ reduction research fields are in their infancy, and knowledge can be learned from each to towards a common goal.

### 1.2. Types of Membranes for CO_2_ Capture and Utilization

**Figure 1:**
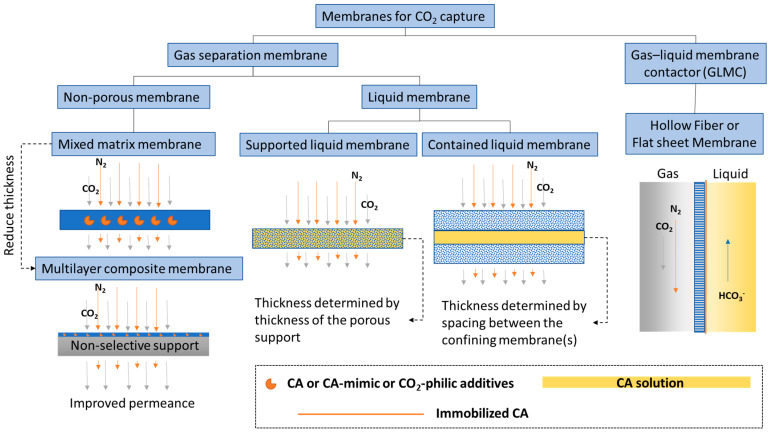
Categories of biocatalytic membranes used for CO_2_ capture.

Membranes are a promising technology platform for CO_2_ capture because they are modular, scalable, and compact. This makes them desirable for process intensification and reducing energy costs [54,55]. Membranes encompass many different types of materials and functionality. In this discussion, to distinguish biocatalytic membranes according to their configurations and separation mechanisms, membranes are loosely divided into two categories, based on the physical states of the fluids separated by the membrane: CO_2_ gas separation membranes and CO_2_ gas–liquid membrane contactors (Figure 1).

#### 1.2.1. CO_2_ Separation Membrane

A membrane that separates two gas phases on either side—CO_2_ lean gas mixture on the feed and CO_2_ enriched gas phase on the permeate side—is called a CO_2_ separation membrane. This category encompasses a large selection of membrane types from non-porous glassy polymer membranes, fixed-site carrier membranes [56], and ultrathin nanocomposite membranes [57], to contained liquid membranes [58]. Research efforts on CO_2_ separation membranes have focused on improving performance-limiting membrane properties, such as CO_2_ gas permeance and selectivity [59]. New classes of polymer materials, such as polymers of intrinsic microporosity (PIM) [60,61] and ladder polymers [62], have been invented that show superior CO_2_ separation properties well above the empirical Robeson upper-bound [63], which classically delineates the trade-off relationship between gas permeability and selectivity. However, physical aging is still an issue that needs to be solved. This problem is common to all glassy polymer membranes, including in the new classes of materials, albeit to a lesser extent owing to the presence of inherent structural porosities. In one case, treatment with super critical CO_2_ altered the internal structure of a PIM, leading to decreased CO_2_ permeance [64]. In another case, after being physically aged, ladder polymers showed increased selectivity but decreased permeability [62], indicating a decreased free volume. To alleviate physical aging issues in glassy non-porous polymer membranes, inorganic aging-resistant CO_2_-philic components are added to the polymer matrix to form mixed matrix membranes (MMM). Recently, Tan et al. [65] discovered a new method for adding high loadings of zeolite into a polyimide membrane matrix that achieved a CO_2_/CH_4_ mixed-gas selectivity of ~423 and a CO_2_ permeability of ~8300 Barrer at moderate pressure and ambient temperature. To put these numbers in perspective, at a similar CO_2_/CH_4_ selectivity of 400, the 2008 Robeson upper-bound for the CO_2_/CH_4_ pair anticipates a CO_2_ permeability of only ~1 Barrer [63].

In order to improve the overall sustainability profile of CO_2_ separation technologies, biopolymer-based MMM, such as chitosan-based non-porous membranes, have recently emerged as alternatives to conventional non-renewable polymer matrices [66]. Casado-Coterillo et al. [67] fabricated a chitosan MMM filled with metal organic framework (MOF) and non-toxic ionic liquid that achieved a high permeability of 4754–5413 Barrer (or 47–52 GPU) and a CO_2_/N_2_ selectivity of 12–19. Borgohain et al. [68] synthesized carboxymethyl chitosan as a matrix for compatibilization with scarcely soluble multi-walled carbon nanotubes (MWCNT) to make a thin MMM selective layer (2.7 µm) that exhibited a CO_2_ permeance of 43 GPU and a CO_2_/N_2_ selectivity of 45. The hydrophilicity and free amine groups of the chitosan material could be contributing to the excellent CO_2_ transport properties, especially in humidified conditions, compared with the commercial hydrophobic membranes [69]. Owing to their abundance in nature, tailorable functional groups, and excellent membrane forming properties, chitosan [70] and other polysaccharides [71,72], could play an increasing role in the fabrication of novel CO_2_ separation membranes.

Another way to improve membrane performance is by making thin film composites (TFC) [73] or integrated multilayer membranes [74], both with ultra-thin CO_2_ selective layers for facilitated CO_2_ transport. CA and CA mimics have been successfully used to construct both MMM and thin CO_2_ selective layers for facilitated CO_2_ separation [75,76]. However, these advanced facilitated transport membranes are still at lab-scale and no direct comparison between these and commercial scale CO_2_ chemical absorption processes is available in the literature. Nevertheless, a recent techno-economic analysis (TEA) study compared a non-facilitated polymeric membrane process (Membrane Technology and Research, Inc., Newark, CA, USA) [77] to an enzyme-based chemical absorption process (Akermin Inc., St. Louis, MO, USA) and found that the latter is economically more attractive in a simulated CO_2_ capture scenario from a 600 MW_e_ power plant flue gas. This result emphasizes the potential for enzymes to improve energy efficiency of conventional energy intensive processes. Interestingly, the study also predicted that the membrane technology could become more efficient if CO_2_ permeance at low pressure (<1.5 bar) could be enhanced. Because CA is already particularly efficient at converting CO_2_ to bicarbonate at ambient pressure, developing low pressure facilitated CO_2_ transport membranes that utilize the fast enzyme reaction rate is a promising concept.

Liquid membranes that separate two gas phases are also defined as CO_2_ separation membranes. CA plays a similar CO_2_ hydration facilitator role in liquid membranes, provided there is water present. General types of liquid membranes include supported liquid membranes and contained liquid membranes (Figure 1). Sometimes distinctions are made between supported liquid membranes (SLM) and immobilized liquid membranes (ILM), where in the first case, liquid fills spaces between fibers in the membrane and the second case, liquid fills specific pores in the membrane [78]. However, most of the time, these two nomenclatures are used interchangeably. Disadvantages of common SLMs or ILMs include the formation of gravity-induced downward bulges in the liquid phase (called catenary curves), low tolerance to transmembrane pressure differences, and a high evaporation tendency. All of these problems can be alleviated by contained liquid membrane configurations in which liquid is bound by porous membrane surfaces [78]. Different types of liquid can be used to construct liquid membranes, including hydrogels [79], ionic liquids [80], deep eutectic solvents [81], and aqueous buffers [82]. Both flat sheet and hollow fiber membranes are commonly used. Considering the variety of configurations and liquid types and the large number of associated publications, liquid membranes are further discussed in a separate section from facilitated non-porous polymer membranes.

#### 1.2.2. CO_2_ Liquid Contactor Membrane

A membrane that separates a gas phase containing CO_2_ from a liquid phase where CO_2_ is absorbed, is categorized as a CO_2_ liquid contactor membrane (Figure 1). This category emerged as a new hybrid membrane system, called gas–liquid membrane contactors (GLMC), that combines the modularity and high surface area of the membrane with the high selectivity of the chemical absorption process [83,84]. Non-enzymatic GLMC developments have focused on improving membrane stability [85], minimizing pore wetting [86], and selecting the best solvent and activator [9]. Reviews of modeling methods used to analyze the mass transfer in hollow fiber gas–liquid membrane contactors (HFGLMC) for post-combustion carbon capture are available [83]. Improvements to membrane materials were also explored by blending polysulfone (PSf) with PEI, a CO_2_-philic polymer. The observed optimal additive ratio for higher capture performance was attributed to chemical affinity, whereas non-optimal conditions inadvertently caused pore wetting and clogging by K_2_CO_3_ precipitation [87].

Another way to improve GLMC performance is increasing the mass transfer of CO_2_ at the gas–liquid interface catalyzed by CA enzymes, which are either immobilized on the membrane [88], dissolved in the solvent [89], or immobilized both on the membrane and on mobile nanoparticles dispersed in the solvent for additional process intensification [90]. A recent TEA study compared a CA-immobilized hollow fiber membrane contactor (HFMC) with benign solvent and vacuum-assisted solvent regeneration with the benchmark case where monoethanolamine (MEA) was used in a conventional packed column process. The projection estimated that at 90% CO_2_ capture from a 685 MW_e_ coal-fired power plant, the enzymatic process achieved a 43% reduction in energy consumption of the capture and compression unit, a 31% reduction in capital cost (CAPEX), and a 28% reduction in operating expenses (OPEX) in comparison with the MEA benchmark [91]. Enzyme-based GLMC is discussed in more detail below.

#### 1.2.3. Other Membrane Structure Functions

**Figure 2:**
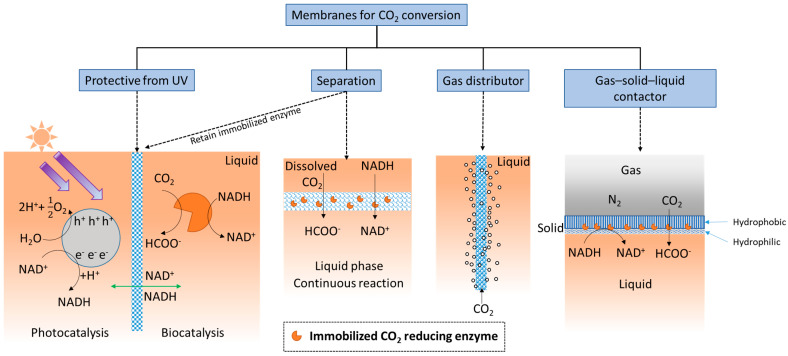
Functionalities of membranes used for biocatalytic CO_2_ conversion.

The simplest definition of a membrane is a thin layer that acts as a boundary or barrier. This barrier can prevent random mass exchange based on size or physical phase, or can provide protection against harsh environments. Membranes used for CO_2_ conversion and utilization applications may require different or added functionality compared with those used for CO_2_ capture. For example, as shown in Figure 2, an ultraviolet (UV) protective membrane was used to block UV irradiation and simultaneously retain enzymes (based on their large size) on the biocatalysis side [92], while allowing small cofactor molecules to freely pass between the separate photocatalytic and biocatalytic reaction chambers.

Additionally, membranes provide ample surface area for enzymes to be immobilized, and therefore, can provide high catalytic enhancement. Considering the membrane’s separation function, when substrate is delivered as dissolved CO_2_-saturated water [35,36], the membrane structure creates a localized environment where the CO_2_ conversion reaction can take place continuously in the liquid phase. Membranes can separate either dissolved or immobilized biocatalysts from products [38,39] (exemplified by two schematics under “Separation” in Figure 2). The importance of this seemingly simple function of solid–liquid separation and recovery of enzymes should not be underestimated. An evaluation of using ultrafiltration membranes to separate dissolved enzymes from a CO_2_-rich solvent [93], to avoid pumping the enzymes into a high temperature desorber for solvent regeneration, found that even with an enzyme retention rate as high as 99.9%, only 50% of the enzymes are retained after 1 month of operation. Therefore, strategies that prevent enzymes from leaching through or away from membranes can be critical. Biocatalyst retention by immobilization is especially important for operating enzymatic membrane reactors for CO_2_ reduction catalyzed by oxidoreductases.

As illustrated under ‘gas distributor’ in Figure 2, porous membranes, specifically porous hollow fiber membranes, can be used to infuse gaseous CO_2_ into the reaction medium [94] to increase the availability of soluble CO_2_. This approach is often used in conjunction with adjacent sets of hollow fiber membranes with immobilized enzymes attached [95]. In addition, since gaseous CO_2_ is attracted to hydrophobic surfaces, amphiphilic membranes functioning as gas–solid–liquid contactors (Figure 2, right schematic) have recently been developed for converting gaseous CO_2_ into water soluble formic acid [96].

Specific examples and the performance of these various membrane functionalities are discussed in detail in Section 5.

## 2. Facilitated Transport Membranes

### 2.1. CA vs. CA-Mimic

Published examples of adding CA enzymes directly into the polymer matrix of a separation membrane are rare. The concerns with this approach include enzyme tolerance to membrane fabrication solvents and quality of protein dispersion in the polymer solution. In one example, Zheng et al. [20] compared Pebax-1657 mixed matrix membrane (MMM) doped with either CA-mimic in the form of a Zinc-coordinated metal organic framework (MOF) or with bare CA enzymes. CA-doped MMM fabricated by solvent evaporation showed a 4- to 5-fold improvement compared with the CO_2_ permeability of the Pebax-1657 control at a pH range of 7–10. The CA-mimic doped MMM demonstrated an 8- to 9-fold enhancement over a wider pH range of 5–11, which was attributed to the CA-mimic’s dual function of resembling the CA enzyme active site and benefiting from a well-defined MOF pore structure for gas transport. In another study, Zhang et al. [75] encapsulated a high loading amount (~24 wt%) of CA enzyme in Zinc-coordinated 2-Methylimidazole metal organic framework (ZIF-8) cavities grown in situ on an oriented halloysite nanotube (HNT) layer supported by a polyacrylonitrile (PAN) porous membrane. The optimal CO_2_/N_2_ selectivity was 166, which was about 21-fold higher than the negative control membranes containing denatured CA. Because the only difference between the two was the activity of the CA, the enhancement was attributed solely to the catalytic effects of the CA enzyme.

**Figure 3:**
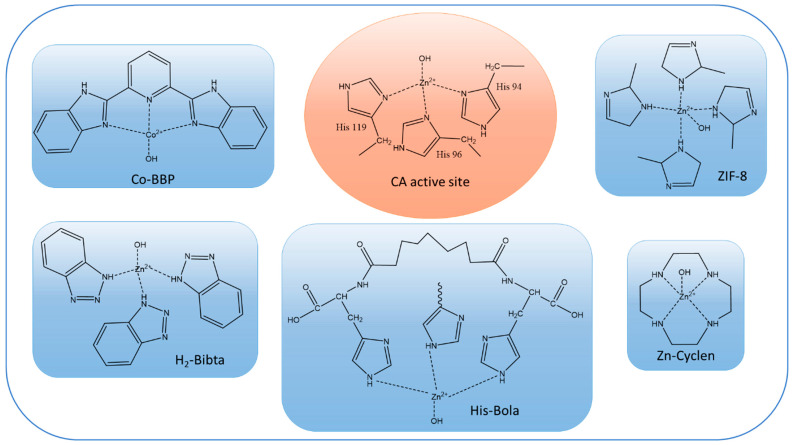
Chemical structures of active sites of CA enzyme (shown in orange oval) and CA-mimics (shown in blue rectangles).

Most of the literature on biomimetic CO_2_ facilitated transport membranes only uses CA-mimics for catalytic enhancement. The most common CA-mimics are Zn–cyclen [76], Cobalt-coordinated Co-2,6-bis(2-benzimidazolyl)pyridine (Co-BBP) [19], Zinc-coordinated histidine-based bolaamphiphile (His-Bola) [97], and Zinc-coordinated bibenzotriazoles (H_2_-bibta) [20]. Comparisons of the active sites of CA-mimics with CA enzymes are provided in Figure 3. Although the reaction rates per active site of the CA-mimics are not comparable with those of the CA enzymes, their lower molecular weights make it possible to load a large number of active sites per unit mass of synthetic polymer membrane, which partially offsets the lower activity. Additionally, high thermal stability (>200 °C) and a wide effective pH range make CA-mimics a popular choice for fabricating facilitated transport membranes [76]. This situation may change as ultrastable CA enzymes [21] become more readily available.

### 2.2. Membrane Structures

Two common pathways to improve CO_2_ separation performance of non-porous membranes are the creation of mixed matrix membranes (MMM) or multilayer composite membranes with a CO_2_ selective layer. The former involves the incorporation of solid CO_2_-philic additives in a continuous polymer matrix, which combines advantages from both materials. The later reduces membrane thickness to improve CO_2_ permeance by supporting a thin CO_2_ selective layer on a less selective but more sturdy gas permeable membrane.

Zhang et al. [19] dispersed 1.33 wt% of CoBBP CA-mimic in Pebax-1657 (PEO:PA6 polyamide 60:40 wt%) matrix through a dissolution–evaporation process for MMM and achieved a CO_2_ permeability of 675.5 Barrer and a selectivity of 62, which is above the empirical Robeson upper-bound. Nilouyal et al. [97] incorporated up to 9 wt% of Zinc-coordinated His-Bola nanoparticles in Pebax-1657 matrix and obtained a higher CO_2_/N_2_ selectivity of 158.2 but a lower CO_2_ permeability of 188.4 Barrer, which also surpassed the Robeson upper-bound. However, after accounting for their thickness of 70–75 μm, these MMMs had CO_2_ permeances in the single digits, at 9 and 3 GPU, respectively. To increase CO_2_ permeance, additional CO_2_ transport mechanisms are needed. Zheng [20] et al. incorporated a CA-mimic that forms MOF porous structures itself and supplements the catalytic function with additional CO_2_ diffusion pathways. Similarly, Wang et al. [98] compounded 28.5 wt% CoBBP CA-mimic with porous organic polymers (POP) that contain inherent microporous structures before mixing them into the Pebax matrix. Both of these examples increased the permeability significantly, with CO_2_ permeances of around 30 GPU at a membrane thickness of 50 μm.

Membrane thickness is a bottleneck for achieving high CO_2_ permeance. The solution is to construct multi-layer composite membranes with a thin selective layer for providing gas selectivity and thicker gas permeable layers as physical supports for providing mechanical stability. For example, Jahan et al. [99] made cast poly(vinyl alcohol) (PVA) MMM containing 1 wt% crystalline nanocellulose and 5 μmol/g Zn-cyclen on a polysulfone (PSf) ultrafiltration membrane. The thickness of the selective MMM layer was 800 nm with a CO_2_ permeance of 126 GPU and a CO_2_/CH_4_ selectivity of 42, which are significantly higher than the prior examples. Saeed et al. [100] used a similar strategy in combining 1 wt% carbon nanotubes (CNT) as nano-filler with 5 μmol/g Zn-cyclen in PVA to create a selective layer with a thickness of 830 nm supported on PSf membrane. Adding CNT resulted in a higher water swelling capability of the PVA membrane and a high CO_2_ permeance of 363 GPU and a CO_2_/N_2_ selectivity of 120.

In special cases, where CO_2_ needs to be separated from smaller gas molecules, such as H_2_ and He, the thickness of the membrane cannot be reduced without affecting CO_2_ selectivity. For example, the CO_2_ permeance of a poly(amidoamine) (PAMAM) dendrimer/poly(ethylene glycol) (PEG) hybrid membrane, which works by a facilitated transport mechanism through amino groups of the PAMAM [101], is lower with an inverse relationship with the membrane thickness (Q_CO_2__ ∝ L^−0.62^), whereas hydrogen permeance has an almost inverse relationship with the thickness (Q_H_2__ ∝ L^−0.95^). Therefore, the selectivity of CO_2_ over H_2_ decreased with a reduced membrane thickness. However, when CA enzyme was spray-coated on the sweep side of the hybrid membrane, the membrane’s CO_2_ permeance became nearly inversely proportional to membrane thickness (Q_CO_2__∝ L^−0.98^), making reducing membrane thickness a possible strategy for improving CO_2_ permeance without sacrificing selectivity. At a membrane thickness of 15 μm, the CO_2_ permeance was 490% higher compared with the no-CA membrane. In this example, rather than distributing CA enzymes or CA-mimics homogeneously throughout the polymer matrix, spray-coating CA enzymes only on the membrane surface made it possible to explore the effect of CA enzyme location on the separation properties. For a fixed membrane thickness, CA spray-coated on the feed side alone did not alter CO_2_ permeance, nor did CA coated on both the feed and sweep sides perform better than CA coated on the sweep side alone. It was, therefore, concluded that the CO_2_ desorption step on the sweep side was the rate limiting step that was assisted by the presence of spray-coated CA enzyme. For membranes that do not contain CO_2_ transport facilitating amino groups, CA coated on both sides of the membrane may be necessary for optimal separation performance. Further studies with different membrane chemistries are needed to fully evaluate the benefits and effects of CA placement relative to the membrane, including on the interfaces and dispersed in the matrix.

### 2.3. Humidity

The mechanism of facilitated CO_2_ transport through bicarbonate formation requires participation of water molecules, regardless of whether it is a CA enzyme [75], CA-mimic [76], or amino group facilitated reaction [101]. For membranes with dual transport mechanisms, the dominating mechanism depends on humidity, and higher humidity favors bicarbonate facilitated transport [20]. As an example, the CO_2_ permeance of the CA-MOF dual-function MMM increased by 62% from dry to humidified conditions [20]. In most cases, both the CO_2_ permeance and selectivity of facilitated transport membranes increase with increasing humidity, and the difference can boost certain membranes to perform CO_2_ separation at above the Robeson upper-bound, even when they otherwise do not achieve this under dry conditions [97]. For context, the empirical Robeson upper-bound was initially constructed based on non-facilitated membrane performance and thus serves as a benchmark for indicating the effectiveness of facilitated mechanisms. Moisture is required for all facilitated CO_2_ transport membranes described here, and the best performance values reported in Table 1 were all obtained under humidified experimental conditions if not otherwise specified.

## 3. Liquid Membranes

Strictly speaking, liquid membranes (Figure 1) promoted by CA and CA mimics are also considered facilitated transport membranes within the gas separation membrane category, where two gas phases are separated on either side of the membrane. However, the fabrication of liquid membranes is distinctly different from the non-porous facilitated transport membranes detailed in Section 2. Often, commercial porous membranes are used to construct liquid membranes, and the choice of either hydrophobic or hydrophilic materials is based on the type of liquids used and the membrane configurations. The history and recent advances of enzyme-promoted liquid membranes are discussed in this section. Their key parameters and performance metrics are summarized in Table 2.

### 3.1. Early Developments in CA-Promoted Supported Liquid Membrane (SLM)

The concept of using CA for enhancing CO_2_ transport through a thin liquid membrane dates back to the late 1960s. Enns [109] demonstrated that transport enhancement across a membrane resulted from a dominating bicarbonate diffusion mechanism because at pH values higher than 6.1, bicarbonate concentration was higher than dissolved CO_2_, and increased bicarbonate concentration was facilitated by the rapid interconversion between dissolved CO_2_ molecules and bicarbonate ions catalyzed by CA. The supported liquid membrane (SLM) used in these tests was created by saturating a Millipore filter with nominal pore size of 0.45 µm, void space of 80%, and a thickness of 150 µm with 25 mM aqueous sodium bicarbonate solutions containing 1–10 mg/mL dissolved CA. In SLM, there is a continuous liquid phase from one side of the membrane to the other. Capillary forces hold the liquid within the open pores of the solid support. CO_2_ transport enhancement of more than two orders of magnitude was possible at pH 9.0 with the highest enzyme concentration. Shortly after, Ward et al. [12] described an “immobilized liquid membrane”, also a flat sheet supported liquid membrane (SLM), in which a porous cellulose acetate film was saturated with 2 N aqueous potassium bicarbonate containing 2 mg/mL of CA. An initial 6-fold enhancement in CO_2_ transport was observed, but this decreased to zero after several days, indicating low stability of the liquid membrane and the CA being used. Therefore, improving the stability of the liquid membrane by reducing solvent evaporation is a primary research objective and requirement for CO_2_ separation applications.

### 3.2. SLM with Non-Volatile Liquids

One of the strategies for retaining enzyme activity and reducing evaporative solvent loss in SLM is the use of non-volatile liquids and thermostable CA strains [110]. Neves et al. [104] chose a hydrophobic ionic liquid supported by a hydrophobic porous PVDF membrane. The addition of 0.01 wt% CA in the ionic liquid showed an enhancement effect only at higher water content, signifying a compromise between optimizing the ionic liquid internal structure and benefiting from enzymatic CO_2_ hydration enhancement. The same was true when a higher CA concentration of 5 mg/mL was added and maximum CO_2_ selectivity versus other gases remained low [80]. Recently, deep eutectic solvents (DES), such as the choline chloride and levulinic acid pair, have been explored to form stable SLM. However, adding CA into DES failed to improve CO_2_ selectivity over N_2_ [81] and in certain cases, decreased its selectivity over CH_4_ [106]. Castro et al. [108] prepared a choline hydroxide-based alkaline solvent containing 14.5% water and 75% glycerol supported on a hydrophilic PTFE microfiltration membrane. With the help of 0.5 mg CA/g solvent and the presence of a large percentage of water, CO_2_/N_2_ selectivity increased to 90.5. Another promising case involves the formation of a stable emulsion for filling the porous membrane. Mondal et al. [107] formulated a water-in-oil emulsion with 1 wt% aqueous disperse phase in corn oil stabilized by 2 wt% Tween 80 and filled the pores of the porous hydrophobic PVDF membrane. The aqueous phase comprised 0.5 g CA per liter potassium carbonate solvent and 5 wt% PEG 300 for enzyme stabilization. With the help of the CA enzyme, CO_2_ permeability increased by ~15% and CO_2_/CH_4_ selectivity increased by 2.9-fold.

### 3.3. CA-Promoted Contained Liquid Membrane (CLM)

As interest in capturing CO_2_ from air and from power plant flue gas grew, new membrane configurations emerged to overcome supported liquid membrane instability. Contrary to SLMs, where hydrophilicity/hydrophobicity of the liquid and support should match in order for the liquid to fill the pores or space in between the fibers of the support [82], contained liquid membranes (CLM) use a mismatch of the hydrophilicity/hydrophobicity of the liquid and the membrane surfaces to create physical barriers that contain the liquid in a confined space (e.g., between flat membrane sheets, or between adjacent hollow fibers (HF)), forming a liquid membrane. Because of the additional physical barriers between the liquid and gas phases, CLMs tend to be less prone to the instability caused by solvent evaporation.

Bao et al. [78] constructed a CLM by surrounding a microporous PP woven HF mat, with mutually orthogonal fiber orientations between hollow fibers containing feed and sweep gases (arranged like a heat exchanger), using different CO_2_ absorption liquids. The CLM with the space between the feed and sweep fibers filled with 1.0 M NaHCO_3_ containing 3 mg/mL dissolved CA performed better than that filled with 20 wt% diethanolamine (DEA). Alternatively, Trachtenberg et al. (Carbozyme Inc., Monmouth Junction, NJ, USA) [58] immobilized CA on the liquid side wall of both feed and sweep hollow fiber microporous membranes in a contained liquid membrane (CLM) configuration to intensify the CO_2_ mass transfer between the gas and liquid and back from liquid to gas at the gas–liquid interfaces. Immobilization alleviated solvent evaporation and enzyme instability, and 85% CO_2_ removal was achieved in a preliminary test using 15.4% CO_2_, a concentration typical for flue gas from coal-fired power plants. Zhang et al. [111] immobilized CA in a poly(acrylic acid-co-acrylamide)/hydrotalcite (PAA-AAm/HT) nanocomposite hydrogel, in the interstitial space between feed and sweep hollow fiber membranes, and reported the immobilized CA retained over 76% activity. However, the bicarbonate diffusion rate through the hydrogel phase was found to be the rate-limiting step of the overall process. The same was concluded about enzyme-based facilitated transport contained liquid membranes (EBCLM), where increases in liquid thickness led to increases in CO_2_ selectivity but decreases in CO_2_ permeance [112].

### 3.4. Liquid Membrane Thickness

Whether it is a SLM or CLM, the thickness of the liquid membrane, which is determined by the thickness of the support membrane or the distance between two surfaces of the support membrane, greatly influences the CO_2_ permeance of the liquid membrane. Typical liquid membrane thicknesses reported in the literature range from 10s to 100s µm, which could result in low CO_2_ permeance even if their permeabilities are high. To decrease the liquid membrane thickness and greatly improve CO_2_ permeance, Fu et al. [105] fabricated an inorganic membrane with close-packed arrays of silica mesopores with a diameter of 8 nm and total thickness (depth) of 1 µm, with the top 18 nm being hydrophilic and the rest being hydrophobic. The hydrophilic portions of the pores were filled with CA solution through capillary forces with an average of 2 CA enzymes per 18 nm thick liquid film in each mesopore. This drastic reduction in liquid layer thickness and high nominal CA concentration inside the channel resulted in a remarkably high CO_2_ permeance of 2600 GPU, and high selectivities of 788 and 1500 for CO_2_/N_2_ and CO_2_/H_2_, respectively, were achieved at room temperature and atmospheric pressure. This example experimentally verified the hypothesis that a reduced liquid layer thickness coupled with high enzyme loading can produce a liquid membrane with outstanding permeance and selectivity. Such encouraging results argue for more studies to demonstrate this phenomenon with other types of support membranes, which will require advancements in respective material processing techniques for fabricating well-controlled nano-sized structures.

## 4. Gas–Liquid Membrane Contactor

### 4.1. Advantages Compared with Conventional Gas Separation Membrane and Chemical Absorption

An important practical challenge for conventional CO_2_ separation membranes lies in the limited partial pressure driving force at atmospheric application conditions. The CO_2_ concentration in the flue gas of a coal-fired power plant is only ~14% and is even lower in natural gas power plant emissions at only ~5% [113]. Both are emitted at near atmospheric pressure, leading to a CO_2_ partial pressure of only 0.05–0.14 bar. Gas permeation occurs when the partial pressure of CO_2_ on the feed side is greater than the partial pressure of CO_2_ on the permeate side:

(3) $$n_{f}\times p_{f}\geq \;n_{p}\times p_{p}$$

where, $n_{f}$ is the molar concentration of CO_2_ in the feed, $p_{f}$ is the feed pressure, $n_{p}$ is the molar concentration of CO_2_ in the permeate, and $p_{p}$ is the permeate pressure. Rearrangement of Equation (3) shows that the maximum separation that can be achieved by a membrane is limited by the ratio of the feed and permeate pressures:

(4) $$\frac{p_{f}}{p_{p}}\geq \frac{n_{p}}{n_{f}}$$

A feed to permeate pressure ratio greater than one is needed to drive the CO_2_ enriching process, no matter how selective the membrane is, and the compression and vacuum cost could easily make the whole process unaffordable [8].

Chemical reactive absorption processes using primary amine solvents have been the benchmark technology for capturing CO_2_ at atmospheric pressure owing to the high CO_2_ removal rate. However, broad adoption of the technology has been impeded by corresponding high regeneration energy consumption (high OPEX) and large equipment size (high CAPEX) [114]. One solution for reducing the high energy penalty is by using lower energy solvents such as sterically hindered amines, tertiary amines, and inorganic carbonate-based solvents [115]. However, the CO_2_ absorption rates of these solvents are often low despite having higher absorption capacity on a mole basis than the primary amine benchmark, monoethanolamine (MEA) [116]. In combination with CA enzymes, however, low energy solvents have the potential to meet MEA absorption efficiency [117].

In addition, the CO_2_ absorption rate can be improved with mass transfer intensification strategies, such as the use of gas–liquid membrane contactors (GLMC), which provide a well-defined interfacial area that is orders of magnitude higher than conventional packed columns [118] along with modularity and a small footprint. Additional advantages of using GLMC stem from its non-dispersive nature which helps avoid common problems in conventional packed columns, such as flooding, foaming, channeling, and entrainment [119]. GLMC modules are typically assembled as a bundle of hydrophobic microporous hollow fibers packed parallel in a shell. Gas and liquid usually flow counter-currently on opposite sides of the membrane (Figure 1). Leimbrink et al. [118] compared the CO_2_ absorption performance of conventional packed column, rotating packed beds, and a GLMC in 30% MDEA, with and without 0.2 wt% CA. Without CA, the three contacting devices had similar CO_2_ absorption per effective interfacial area. With CA, although the membrane contactor showed lower enhancement from the CA enzyme (due to the large specific surface area of the membrane contactor making it seem less effective from enzyme enhancement), it achieved the highest CO_2_ absorption per contactor volume, requiring only a quarter of the size to achieve the same absorption required for a packed column process.

### 4.2. Developments in CA-Promoted GLMC

Interest in improving the CO_2_ removal rate of artificial lung devices prompted the early development of enzyme immobilized GLMC for CO_2_ transport. In contrast to conventional GLMC used for CO_2_ absorption, the essential function of an artificial lung is to act similar to a desorber contactor, where CO_2_, delivered as a liquid flow in the form of dissolved bicarbonate ions, is quickly converted to CO_2_ gas and carried away by the oxygen sweep gas. The oxygen sweep gas acts as the low CO_2_ partial pressure driving force for desorption. This desorption reaction is catalyzed by CA immobilized at the surface of the hollow fiber membranes. Federspiel and coworkers [13,120,121,122,123] utilized plasma-based surface modification techniques to introduce hydroxyl or amino functional groups on hollow fiber membranes (HFM) followed by surface chemical activation using cyanogen bromide or glutaraldehyde, both of which are reactive toward lysine amino groups in CA. Enhancements of 115% and 37% in CO_2_ removal rates were observed from buffer and blood, respectively, after optimizing surface treatment and enzyme immobilization conditions, including the use of chitosan as a tether polymer. Further increases in enzyme activity, by use of a highly active recombinant human CA, did not yield additional improvement to the CO_2_ removal rate due to the lack of sufficient driving force at physiological conditions for CA to push the reaction toward bicarbonate dehydration at the fluid boundary layer. This emphasizes that CA offers a benefit when the reaction is kinetically limited in the presence of a driving force but does not overcome equilibrium dominated conditions. However, when the local equilibrium was altered by introducing acidity at the boundary layer by using an acidic sweep gas, a 109% increase in the CO_2_ removal rate was achieved while still maintaining the bulk blood at physiological pH. This further emphasizes that altering the local equilibrium is a fundamental strategy by which enhanced CO_2_ mass transfer can be achieved in the presence of CA. While GLMC has only been explored somewhat for CO_2_ stripping [124,125], advances in artificial lungs with immobilized CA on HFM could potentially provide alternatives to the high temperature CO_2_ stripping process which consumes the most energy in conventional chemical absorption processes.

The research field of CA-immobilized GLMC for CO_2_ absorption is expanding [126] as more attention is placed on the negative effects of greenhouse gas emissions on our environment and efforts increase to find more efficient and sustainable solutions to mitigate this issue. Representative research studies with their materials selection and key performance outcomes are summarized in Table 3. The dominant research themes include membrane surface modification, enzyme immobilization, absorption solvent development, and reducing mass transfer barriers.

### 4.3. Materials and Surface Modifications

The role of a membrane in GLMC is to separate the liquid phase from the gas phase and provide well-defined non-dispersive contacting areas between the two phases. Therefore, the most commonly used type of membranes are hydrophobic porous membranes, including, poly(methyl pentene) (PMP) [13], polypropylene (PP) [88], and polyvinylidene fluoride (PVDF) [134]. Serving the same function, tubular porous glass membranes can also be used after applying hydrophobic coatings on the outer membrane skin [130]. Surface modifications, such as coating with sol-gel TiO_2_, have been implemented on PVDF membranes to improve enzyme compatibility [127]. However, decreased water contact angle and liquid entry pressure were observed, and severe pore blockages occurred after multiple sol-gel coating cycles. Superhydrophobic coating, 1H,1H,2H,2H-perfluorodecyltriethoxysilane (PDTS), was applied on a TiO_2_ functionalized PP membrane, leading to improved operational stability while inadvertently also increasing membrane resistance [129].

In addition to deliberate surface modifications carried out by chemical reagents and polymer coatings, the surface properties of membranes, including the parts of pores exposed to enzyme immobilization solutions, are drastically altered by immobilized enzymes, often leading to membrane pore wetting. In one study, at a high enzyme loading, the surface pore openings were narrowed and the water contact angle of a pristine PP membrane dropped from 131° to 78° after enzyme immobilization [90]. However, the extra mass transfer resistance caused by partially wetted pores was overcome by enzymatic CO_2_ hydration enhancement caused by CA enzymes attached inside the wetted pores [137]. A biocatalytic GLMC operated in fully pore-wetted conditions has been described where CA was adsorbed on hygroscopic and catalytic MOF grown on the surface of a hydrophilic Al_2_O_3_ membrane, where a CO_2_ hydration rate of 108 μmol cm^−2^ min^−1^ from 5% CO_2_ gas into water was achieved [136], but the buffering capacity of the system was not explained. The additional mass transfer resistance of the wetted pores was likely overcome by the combined catalytic hydration enhancements from CA and MOF. A recent modelling study concurred with the observation that mass transfer resistance in wetted pores can be reduced by catalyzed CO_2_ hydration [138].

To overcome pore wetting caused by immobilized enzymes, a “Janus” configuration was used to modify a PVDF membrane, with hydrophilic carbon nanotubes (CNT) on one side and superhydrophobic fluorosilane on the other side of the membrane [128]. CA was immobilized through physical adsorption onto the hydrophilic side to prevent interference with the pore structure and properties of the hydrophobic membrane. In a pressure drop test, where the hydrophilic side faced water and the superhydrophobic side faced the overhead gas chamber filled with 100% CO_2_ gas to 1 atm, the CA immobilized membrane achieved up to a 2-fold increase in CO_2_ hydration rate compared with the no-enzyme control membranes. The advantage of immobilizing CA on a Janus membrane located at the gas–liquid interface was demonstrated by the fact that over 30 mg of free CA had to be dissolved in the liquid bulk to achieve a similar CO_2_ hydration rate as 2.97 mg CA immobilized on the membrane. Because the mass transfer at the gas and liquid interface is the rate-limiting step, the immobilized enzyme concentrated at the interface was able to catalyze the CO_2_ hydration more efficiently.

In addition to hydrophobic porous membranes, non-porous CO_2_ permeable membranes were also explored as GLMC for CO_2_ absorption. CO_2_ absorption into 30% K_2_CO_3_ solvent using a non-porous polysulfone (PSf) HFM coated with a layer of PDMS [132] was 70–90% that of a porous PP HFM, both incorporating immobilized CA through layer-by-layer electrostatic adsorption [131]. A 60-µm-thick free-standing non-porous PDMS membrane used in microfluidic devices to separate anesthesia gases from an ionic liquid (IL)-based CO_2_ absorption solvent exhibited a 1.9-fold increase in CO_2_ affinity when 0.1 mg CA/ g IL was added, while Xenon permeability was not affected [133]. A seemingly counter intuitive example showing promise was based on a porous hydrophobic PP membrane with a dense non-porous hydrophilic PVDF skin that was surface-coated with a CO_2_ selective, hydrophilic, and anti-fouling poly(ionic liquid) (PIL) layer facing the flue gas side [89]. The added resistance from the hydrophilic PVDF dense non-porous skin layer was insignificant compared with the improved affinity of CO_2_ brought about by the PIL layer, which resulted in a synergy with dissolved enzyme in the solvent and an overall improvement in CO_2_ absorption.

These encouraging examples show that studies on modification of the membrane surface with control over hydrophilicity/hydrophobicity and the location of the immobilized enzymes and CO_2_-philic additives will continue to evolve in positive directions. At the current stage, larger scale demonstration studies are urgently needed to prove the feasibility and longevity profiles of enzymatic GLMC processes for commercialization.

### 4.4. Enzyme Immobilization

The basic principles of enzyme immobilization outlined in the introduction are applicable to membrane immobilization. For GLMC applications, enzymes are exclusively immobilized on the surface of the pre-made carriers, either stationary on the membranes [131,134] or mobile on nanoparticles that are dispersed in the absorption solvent [90,129]. Because GLMCs are positioned at the gas–liquid interface where the limiting step in mass transfer occurs it makes sense to expose the immobilized enzyme as close to this interface as possible. This requires enzymes to be adsorbed to or immobilized on the liquid-facing membrane side or requires enzymes to stay mobile in the liquid and thus have chances to approach the gas–liquid interface as the liquid flows. These principles were combined in a recent report [135] where CA enzymes were immobilized on an electrospun poly(styrene-co-maleic anhydride) PSMA nanofiber membrane that was floated at the air–liquid interface assisted by 3D printed flotation devices, and the actual air and liquid interface was refreshed frequently as the liquid was agitated. Such a configuration could be useful outside of the column-based absorption systems, such as for enhancing CO_2_ uptake in natural systems such as in ponds and lakes.

One important parameter that studies across the field strive to improve is the enzyme loading. Enzyme loading is commonly reported as the mass of enzyme (µg or mg) or esterase activity (U) of CA, either as a total number for the module assembly [13] or divided by the nominal area of the membrane [136] or volume of the reactor [90]. Various strategies can be used to increase enzyme loading. For example, enzyme loading increased linearly with the number of alternating enzyme layers in a layer-by-layer (LbL) electrostatic adsorption technique [131,132]. Enzyme loading can also be optimized by moderating chemical reagent ratios to control surface functional group density [134] or by optimizing instrument power and duration settings, such as plasma radio frequency glow discharge [120,121,122,123]. A monolayer of enzyme coverage, estimated through geometrical calculations, can be compared with the obtained enzyme loading [13]. However, beyond a certain point, the CO_2_ hydration rate of the immobilized enzyme no longer follows a linear correlation with the total amount of enzyme detectable by the esterase activity assay [128]. This is because only the surface-exposed enzymes are able to catalyze the extremely fast CO_2_ hydration reaction, while enzymes buried deeper under the surface are mass transfer limited and, therefore, not able to contribute to the catalytic effect. Nevertheless, higher enzyme loading could indeed improve product longevity because fresh layers of enzyme could be exposed over time to continue the catalytic enhancement [128].

It is widely acknowledged that the immobilized enzyme orientation on surfaces can affect its activity, and the impact of such effects with immobilized CA enzymes have already been studied on simple geometries such as ultra-flat template-stripped gold (TSG) [139]. However, due to the more complex geometry of membranes and the non-specificity of chemical bonds or physical interactions involved in many immobilization approaches, experiments on the controlled orientation of immobilized CA on membranes and the effect on CO_2_ absorption performance have not yet been reported. This is an important research direction as the fields of enzyme immobilization and protein engineering converge toward orienting immobilized enzymes with enhanced activity and stability.

### 4.5. Solvents and Form of Substrate

Initial evaluations of biocatalytic GLMC measured the enzymatic CO_2_ hydration rate enhancement with the membrane immersed in CO_2_-saturated buffers [88,127]. However, this type of configuration circumvented the critical and rate-limiting mass transfer step of CO_2_ from the gas phase to the liquid phase, which is a critical performance function of GLMC that should be evaluated. More realistic configurations, with membranes positioned at gas–liquid interfaces, were later implemented with 100% CO_2_ gas [128] and soon after with more realistic lower concentration CO_2_ gas mixtures [129].

Common liquids used as CO_2_ absorption solvents for lab scale testing are water [136] and low concentration aqueous buffers [137]. The advantages of using these solvents, include abundancy, non-corrosiveness, and less tendency to wet the hydrophobic membrane. However, a drawback is their low CO_2_ capture capacity, which inevitably requires the use of higher amounts of liquid to absorb more CO_2_. Concentrated aqueous solutions of potassium carbonate are relatively benign and are known to both benefit from CA kinetic enhancement and have a useful carbon loading capacity [14]. An aqueous 30 wt% K_2_CO_3_ solvent was used in an HFGLMC apparatus where three layers of CA were electrostatically immobilized, resulting a three-fold improvement in CO_2_ absorption rate compared with the non-enzyme control membrane [131,132]. The immobilized CA retained over 80% activity after a 200 h exposure to common contaminants from flue gas and to the high pH environment brought about by the high solute concentration.

The low regeneration energy solvent 30% methyldiethanolamine (MDEA) was compared with the benchmark solvent 30% monoethanolamine (MEA), both with 1 wt% dissolved CA concentrate, in an HFGLMC configuration [89]. Dissolved CA contributed up to a 2.2-fold CO_2_ absorption rate enhancement in 30% MDEA, while showing a negative effect for 30% MEA. The explanation is that MEA reacts directly with CO_2_ to rapidly form carbamate molecules, which is not enhanced further by CA, which instead acts through a bicarbonate formation mechanism. A less common category of absorption solvent for GLMC, ionic liquid, was also studied with dissolved CA [133]. The water content was essential for detecting an enzyme enhancement effect because the hydration reaction requires 1 mole of water per mole of CO_2_ converted to bicarbonate.

In summary, CA-enhanced GLMCs, with both immobilized and dissolved CA, offer rate enhancement benefits from the biocatalyst and, thus, are able to use aqueous benign solvents, which greatly reduces membrane wetting and improves membrane longevity. Because pumping and heating large volumes of water take a lot of energy, future studies that couple the CO_2_ capture in natural liquid sources with direct utilization have the potential to avoid the desorption cost and make this process more desirable.

## 5. Enzyme Membrane Reactor

Beyond CO_2_ capture, membranes play a vital role in the design of efficient enzyme membrane reactors (EMR) for carrying out CO_2_ utilization chemical conversion reactions. These processes benefit from the combined separation function of the membrane together with continuous-flow chemical reactors. Discussions in this section focus on intense research on the design and application of EMR for CO_2_ reduction reactions that incorporate oxidoreductase enzymes and representative studies are summarized in Table 4.

### 5.1. Location of the Immobilized Enzymes

Biocatalyst stability, recovery and reuse are improved by immobilizing enzymes on membrane supports, utilizing both their high surface area and separation functions. Common immobilization strategies involve modifying porous membrane surfaces using UV-grafting [141], acid [142] or base [140] treatment, silanization [140], polydopamine (PDA) and PEI deposition [145]. The carboxylic acid or amine surfaces are further activated with carbodiimide coupling reagent [95] or glutaraldehyde [142], respectively, rendering the surface covalently reactive toward amino groups on the enzyme. Enzymes immobilized on membrane surfaces have alleviated mass transfer limitations and thus higher activity.

Leveraging the separation function of a membrane, direct membrane fouling [37], also known as dead-end filtration [143], has been a popular non-chemical alternative for enzyme immobilization. Enzyme cascades can be easily constructed by direct membrane fouling methods. Luo et al. [37] immobilized formate dehydrogenase (FDH), formaldehyde dehydrogenase (FaldDH), and alcohol dehydrogenase (ADH), both as a co-localized mixtures and as separate layers, in sequences using direct membrane fouling techniques. While the direct cascade concept of reducing product inhibition and enhancing substrate shuttling is appealing, the presence of a bottleneck enzyme in the cascade, FaldDH in this case, requires a threshold formic acid concentration to move the reaction in the reduction direction, making co-localization less effective. Rather, a cascade consisting of separate enzyme layers is more conducive to higher final product yield because of the flexibility this gives in optimizing reaction steps separately. Zhu et al. [143] encapsulated individual enzymes in metal organic frameworks (MOF) and loaded them either randomly or in sequence into the pores of a support membrane by dead-end filtration. Consistent with the foregoing study, they found that the ordered multi-enzyme cascade system achieved the highest methanol yield.

Another aspect of localizing enzymes by immobilization is to protect enzymes from harsh environments (Figure 2). Kurayama et al. [92] used a ceramic membrane to separate the enzymatic reaction step from the photocatalytic chamber used for cofactor regeneration. They found that when ultra-violet (UV) light shone on the whole system and directly on the enzymes, no formic acid production occurred, indicating the critical importance of the UV blocking function. Alternatively, Guo et al. [94] separated the NADH-depleted liquid and pumped it to a separate quartz vessel for photo-regeneration, away from the enzymatic reaction chamber. Tian et al. [148] encapsulated enzyme in zeolitic imidazolate-based MOF to protect FDH from possible deactivation by reactive oxygen species (ROS) generated in the photocatalytic reaction. Chai et al. [146] used thermal stable MOFs as protective coatings to encapsulate FDH and protect it from high temperature flue gas conditions. While the free enzyme denatured immediately, the MOF encapsulated enzyme maintained activity even after 4 h at 100 °C. However, they pointed out that the NADH cofactor has larger dimensions than the pore sizes of the MOF, thus limiting the effectiveness of the immobilized enzymes to those exposed at the surfaces. This issue was investigated in another study [143], where FDH, FaldDH, or ADH were paired with and co-immobilized with glutamate dehydrogenase (GDH) for enzymatic NADH regeneration. The methanol production rate increased in the cases where NADH was also co-immobilized in the MOF structures. It seems that although the well-defined native MOF pores do not fit the large NADH, the pre-encapsulated NADH generated larger cavities that were able to accommodate it for a faster reaction.

### 5.2. Roles of Membrane in Substrate Uptake

Due to its low aqueous solubility, delivering CO_2_ to the active site of enzymes becomes a rate-limiting step. For many of the early studies, CO_2_-saturated water or buffer [37,92] was used to circumvent the gas to liquid mass transfer limitation. For continuous delivery as a gas, CO_2_ was bubbled into the reaction chamber [142]. CO_2_ gas can also be infused into the liquid by using hollow fiber membranes (HFM) as a CO_2_ gas distributor [95], utilizing both the high surface areas and porous structures of the membranes to generate small gas bubbles for faster dissolution. For batch reactions, CO_2_ can be blown into the liquid and pressurized in the head space to a certain pressure before sealing the reaction chamber [145].

Another strategy to increase CO_2_ uptake in the reaction solution, borrowing knowledge from the CO_2_ reactive absorption process, is the use of amine-based CO_2_-philic materials. Wang et al. [141] found that a PEI modified HFM surface was able to bring in more CO_2_ from the gas phase in the form of bicarbonate ions and improve formic acid production of dissolved FDH. This implies that CA could also assist FDH to reach a higher productivity. Chai et al. [146] constructed a GLMC with immobilized CA and FDH, combining CO_2_ capture with CO_2_ conversion in a single step. By positioning the GLMC at the gas–liquid interface with the hydrophilic side facing the liquid, the biocatalytic membrane produced 5.6 μmol formic acid after 4 h of reaction using 20% CO_2_ gas.

In addition, the hydrophilicity and hydrophobicity of the membrane have an influence on CO_2_ gas accessibility to the enzyme. Lin et al. [147] measured the CO_2_ gas bubble adhesion force on both hydrophobic and hydrophilic membranes and concluded that hydrophobic membranes attracts CO_2_ gas bubbles better. FDH immobilized on a hydrophobic layer achieved higher formic acid yield than that immobilized on a hydrophilic layer.

### 5.3. Cofactor Regeneration

While many studies used the natural cofactor NADH as the electron donor [95,140,141,145,146] as a proof-of-concept, the continuous supply of NADH to run the reaction can be costly and is uneconomical for real-world CO_2_ reduction applications. To overcome this limitation, NADH regeneration has been the focal point of many recent research publications. There are three main approaches for NADH regeneration, namely, biocatalytic [143], photocatalytic [147], and electrochemical [142].

Biocatalytic regeneration involves the use of another enzyme and its substrate as a natural reducing electron source to reduce NAD^+^ to NADH. Zhu et al. [143] co-immobilized glutamic dehydrogenase (GDH) with FDH, FaldDH, and ADH. A continuous supply of relatively inexpensive L-glutamate as the reductant powered the enzymatic cascade reduction reactions from CO_2_ to methanol.

Photocatalytic regeneration uses semiconductors or organic dye-based photosensitizers to harvest light energy for creating high energy electrons that can be transferred to NAD^+^ for its reduction. However, direct electron transfer from the photosensitizer to NAD^+^ is inefficient, and an electron mediator is usually needed. Kurayama et al. [92] paired TiO_2_ particle photo-catalysts with oxidized methyl viologen (MV^2+^) as the electron mediator with the enzyme diaphorase (DAH) that accepts the reduced form of MV^+^ as the reducing equivalent in reducing NAD^+^ to NADH. The results showed that the concentrations of both MV and NADH affected the overall CO_2_ conversion rate, and that the DAH catalytic reaction was the rate-limiting step. The requirement of an additional enzyme to transfer the reducing equivalents from an electron mediator to NAD^+^ is disadvantageous as it adds additional bottlenecks, such as the DAH catalytic process.

Electrochemical approaches aim to overcome cofactor-dependent reaction limitations. A Rhodium-based compound named pentamethylcyclopentadienyl rhodium bipyridine complex, [Cp⁎Rh(bpy) (H_2_O)]^2+^, was reported to directly interact either with nicotinamide cofactors or with a reagentless source of reduction equivalents, such as a cathode [149]. Gu et al. [144] employed soluble homogenous [Cp⁎Rh(bpy) (H_2_O)]^2+^ along with a heterogeneous TiO_2_ photo-catalyst and achieved a maximum turnover number of 125 after 4.5 h for NADH regeneration. Guo et al. [94] utilized a similar regeneration system for the synthesis of formaldehyde from CO_2_ with both FDH and FaldDH immobilized on hollow fiber membrane. They compared the pH and sacrificial electron donor and found that ethylenediaminetetraacetic acid (EDTA) produced much higher formaldehyde yield than water over a pH of 5–7.5.

**Figure 4:**
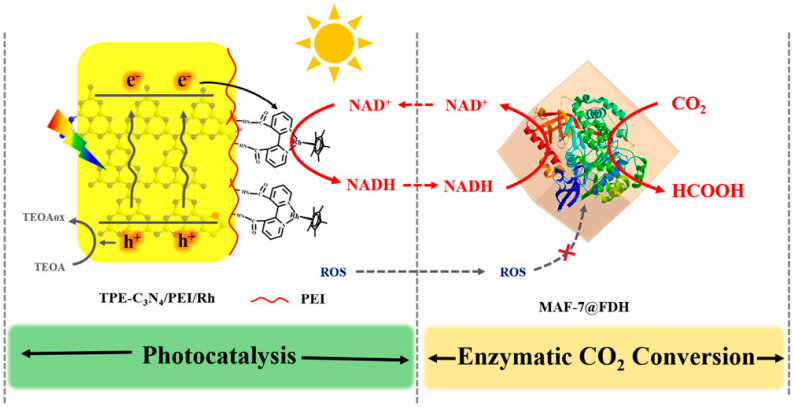
Modified graphitic carbon nitride decorated with a Cobalt complex for photocatalytic NADH regeneration (Reprinted with permission from Ref. [148]. Copyright 2020, American Chemical Society).

The problem with using homogeneous (dissolved) electron mediators is that they travel with the reaction liquid throughout the system and thus require a large amount of mediator to be used to achieve an effective concentration. The solution to this is to confine or immobilize the electron mediator in a localized domain. Tian et al. [148] constructed an elaborate compartmentalized photocatalyst-enzyme system inspired by the thylakoid membrane (Figure 4). The electron–hole pair separation ability of an inorganic photosensitizer graphitic carbon nitride C_3_N_4_ was improved by incorporating aromatic thiophene into network structures, and the selective electron transfer to NAD^+^ was mediated by a Cobalt complex coordinated with bipyridine covalently bonded to the surface modifier polyethylenimine (PEI) for efficient NADH regeneration. Lin et al. [147] synthesized a Ti-based MOF with an electron-transferring Rh complex anchored to the light-harvesting iminopyridine unit of the MOF and found an NADH generation yield of 66.4% in 60 min. Zhang et al. [96] covalently grafted a Rh complex onto amino-functionalized polymeric carbon nitride photo-catalyst for efficient regeneration of NADH with a conversion of 66% in 20 min.

A common drawback for all photocatalytic systems is the use of a sacrificial electron donor, after photo excitation and electron transfer, to deliver electrons to fill positively charged holes and regenerate the photo-catalysts. This electron donor can be abundant compounds, such as water, but the most efficient ones, such as EDTA and triethanolamine (TEOA), would cause additional cost and chemical waste issues. Recently, some metal-dependent FDHs have been reported to accept artificial electron donors by mediated electron transfer (MET) [150], circumventing the use of the NADH cofactor. Obtaining electrons directly from electrodes through direct electron transfer (DET) is an appealing concept but it usually comes at the cost of lower interfacial electron transfer rates due to the need for direct contact between electrode and enzyme, thus limiting effective enzyme loading to only a monolayer [151]. However, as of now, neither of these NADH-free mechanisms has been used in conjunction with membrane technologies, and future work on utilizing high surface area conductive membranes could potentially overcome the enzyme loading limitation and result in highly efficient integrated electrochemical-enzymatic CO_2_ reduction technologies.

### 5.4. Long-Term Stability of EMR

The long-term stability of EMR includes storage and operational stabilities, and both need to be addressed before large scale-up and commercialization is possible. A consistent finding across studies is that the storage stabilities of immobilized enzymes are greatly improved over their free dissolved enzyme counterparts. For example, FDH covalently attached on HFM retained 83% and 67% activity after 30 and 60 days, respectively, in buffer at 4 °C, compared with 48% activity retained for the dissolved FDH after only 14 days under those conditions [95]. FDH immobilized on electrospun polystyrene nanofiber retained 41% activity after 20 days storage in buffer at 4 °C [142], and close to 100% activity retention was reported for CA and FDH encapsulated in MOF membranes after 20 days storage in ambient air [146]. Most studies report operational stabilities as the “number of recycles”, which is usually limited to 10–20 cycles with total operation times of several to tens of hours [37,142]. For example, ADH immobilized on ceramic silicon carbide membranes retained less than 20% of the initial activity after 17 cycles of reuse [140]. However, high activity retention over many days of operation will be needed to fully validate the operational stability of EMRs. Lin et al. compared FDH immobilized either on the hydrophobic support layer or the hydrophilic skin layer of a photo-biocatalytic membrane system over five 24 h cycles of operation (120 h total) and found that although the former generated more formic acid, both configurations were able to retain all of their initial productivity at the end of test [147]. Guo et al. comprehensively tested operational stability by running their reaction continuously for 48 h, finding that a photo-enzyme coupled system was significantly more stable than photocatalysis (UV/TiO_2_ reduction of CO_2_) or biocatalysis (FDH and FaldDH without NADH regeneration) alone [94]. This type of continuous operation testing is highly important in future studies. Analogous to the CO_2_ reduction reaction, the enzymatic fuel cell field has long been battling with short lifetimes of bio-electrodes and instabilities of electron mediators [152]. By carefully fine tuning the enzyme immobilization matrix, lifetimes of more than 45 days or even 200 days of continuous operation are possible [153,154,155]. Certainly, the experiences [156,157] gained in biofuel cell research should be adopted for the fabrication of stable biocathodes for CO_2_ reduction reactions, and we expect more long-term stability tests will be carried out with this goal.

## 6. Conclusions and Future Perspectives

Biocatalytic membranes are a promising technology category for CO_2_ capture and utilization that combine high reaction rates and enzyme selectivity with high surface area and separation functions of the membrane. CA and its mimics have been used in both immobilized and in dissolved forms in conjunction with membranes, where the presence of water molecules is essential for the facilitated CO_2_ transport mechanism to function, regardless of the membrane type. For mixed matrix membranes and liquid membranes, membrane thickness is the bottleneck for achieving high CO_2_ permeance. Recent trends in constructing ultra-thin CA immobilized selective layers or liquid-immobilizing porous structures, both supported by mechanically stable non-selective layers, for high permeance CO_2_ separation, are expected to continue as more sophisticated fabrication methods are devised for reducing the membrane thickness.

CA-enhanced gas–liquid membrane contactors (GLMC), with both immobilized and dissolved CA, offer rate enhancement benefits for the biocatalyst and, thus, are able to use benign aqueous solvents, which greatly reduces membrane wetting and improves membrane longevity. Although issues such as enzyme-induced membrane wetting, surface hydrophilicity and pore blockages can occur, the additional mass transfer resistance of the wetted pores can still be overcome by the catalyzed CO_2_ hydration. Because the mass transfer at the gas–liquid interface is the rate-limiting step, immobilized enzymes concentrated at this interface are able to catalyze the CO_2_ hydration reaction more efficiently. Modifications of membrane surfaces with increased control over hydrophilicity/hydrophobicity and the location of immobilized enzymes and CO_2_-philic additives will continue to evolve and improve reaction performance. In addition, only limited exploration of GLMC for CO_2_ stripping has occurred. Advances in artificial lungs with immobilized CA on hollow fiber membranes (HFM) could inspire alternatives to high temperature CO_2_ stripping processes which are responsible for high energy consumption.

While it is widely acknowledged that the orientation of enzymes on surfaces affects the activity of immobilized enzymes, complex membrane geometries and non-specificity of chemical bonds or physical interactions involved in many immobilization approaches have hindered experiments on controlled orientation of immobilized CA on membranes. Thus, the impact of oriented CA on CO_2_ absorption performance is yet to be reported. This is an important research direction as the fields of enzyme immobilization and protein engineering converge toward orienting immobilized enzymes with enhanced activity and stability. Likewise, enzyme cascades for CO_2_ conversion, currently constructed by direct membrane fouling, would benefit from improved control over enzyme placement and orientation. The presence of a bottleneck enzyme in the cascade requires a minimum threshold substrate concentration to move the reaction in the reduction direction, making co-localization of enzyme pairs potentially less effective, though controlling the molar ratio of enzymes in proximity to each other may help alleviate this issue. Alternatively, cascade reactions consisting of separate enzyme layers could achieve higher final product yields, due to the corresponding flexibility in optimizing each separate reaction step.

Poor aqueous solubility of CO_2_ is a rate limitation for delivering CO_2_ to enzyme active sites for CO_2_ conversion. Utilizing CA-promoted CO_2_ hydration reactions together with HFM CO_2_ gas infusion is one way to supply higher concentrations of carbon in the form of bicarbonate to solute selective membranes or to CO_2_ reducing enzymes. Furthermore, coupling CO_2_ capture in natural liquid sources with direct utilization has the potential to completely avoid desorption costs and make this process more desirable. New gas–liquid–solid tri-phase contactor configurations have the potential to further integrate and improve the capture and conversion processes.

A critical challenge in the application of CO_2_ reducing enzymes for CO_2_ conversion is the delivery of reducing equivalents, i.e., electrons and protons. While methods to regenerate the natural cofactor NADH have been the focus of many studies in this field, and proofs-of-concept for biocatalytic, photocatalytic, and electrochemical NADH regeneration methods have been reported, more research is needed to develop and scale up these important processes. In other areas of biocatalytic reactor research, certain metal-dependent FDHs are reported to accept artificial electron donors by mediated electron transfer (MET) or obtain electrons directly from the electrode through direct electron transfer (DET). Since neither of these NADH-free mechanisms has yet been used in conjunction with membrane technologies, future work on utilizing high surface area conductive membranes should be conducted. Such efforts could potentially overcome enzyme loading limitations and result in highly efficient integrated electrochemical-enzymatic CO_2_ reduction technologies that are urgently needed to address the climate change crisis.

## Figures and Tables

**Table 1:** Facilitated transport membranes with carbonic anhydrase or enzyme mimics for CO_2_ separation.

Application	Membrane Configuration	Enzyme, Concentration	Performance	Year, 1st Author, Ref
CO_2_ separation from N_2_	PVA selective layer containing enzyme mimic supported by PSf ultrafiltration membrane	5 μmol/g (Zn–cyclen /PVA) with 1 wt% CNT	CO_2_ permeance: 256–363 GPU CO_2_/N_2_ selectivity: 107–120	2015–2016 Saeed [76,100]
CO_2_ separation from N_2_	Biocatalytic composite membranes HNTs/MOF/CA selective layer supported by PAN membrane	24.2 wt% CA in MOF	CO_2_ permeance: 24.2 GPU CO_2_/N_2_ selectivity: 165.5	2017 Zhang [75]
CO_2_ separation from N_2_	MMM with Cobalt-based CA-mimic CoBBP dispersed in Pebax-1657 (PEO:PA6 polyamide 60:40 wt%)	1.33 wt% CoBBP in Pebax	CO_2_ permeability: 675.5 Barrer CO_2_ permeance: 9 GPU (75 μm thickness) CO_2_/N_2_ selectivity: 62	2018 Zhang [19]
CO_2_ separation from H_2_	PAMAM/PEGDMA/GMA hybrid membrane supported on PES porous support	1 wt% CA loading spray-coated on the hybrid membrane	CO_2_ permeance: 14.4 GPU CO_2_ permeability: 216 Barrer (15 μm thickness) CO_2_/He selectivity: 28.7	2019 Duan [101]
CO_2_ separation from N_2_	MMM with CoBBP CA-mimic loaded on POP (CoBBP@POP) and together both were loaded in Pebax-1657 matrix	28.5 wt% CoBBP in POP. 5 wt% CoBBP@POP composite in Pebax matrix.	CO_2_ permeability: 1620 Barrer CO_2_ permeance: 32.4 GPU (50 μm thickness) CO_2_/N_2_ selectivity: 102	2020 Wang [98]
CO_2_ separation from CH_4_	PVA selective layer containing CA-mimic supported by PSf ultrafiltration membrane	5 μmol/g (Zn–cyclen /PVA with 1 wt% CNC)	CO_2_ permeance: 126 GPU CO_2_/CH_4_ selectivity: 42	2021 Jahan [99]
CO_2_ separation from N_2_	MMM with His-NPs CA-mimic loaded in Pebax-1657 matrix	0–9 wt% His-NPs in Pebax-1657 matrix	CO_2_ permeability: 188.4 Barrer CO_2_ permeance: 2.7 GPU (70 μm thickness) CO_2_/N_2_ selectivity: 158.2	2022 Nilouyal [97]
CO_2_ separation from N_2_	MMM with Zinc-coordinated MOF CA-mimic loaded in Pebax-1657 matrix	3% MOF CA-mimic in Pebax-1657	CO_2_ permeability: 869 (dry) Barrer 1409 (humid) Barrer CO_2_ permeance: 28.2 GPU (50 μm thickness) CO_2_/N_2_ selectivity: 88.6 (dry) 83 (humid)	2022 Zheng [20]

Acronyms: PVA—poly(vinyl alcohol); PSf—polysulfone; GPU—gas permeation unit: 10^−6^ cm^3^(STP)/(cm^2^ s cm Hg); CNT—carbon nanotube; PAN—polyacrylonitrile; HNT—halloysite nanotube; MOF—metal organic frameworks; CA—carbonic anhydrase; MMM—mixed matrix membrane; CoBBP—Co-2,6-bis(2-benzimidazolyl)pyridine; PEO—poly(ethylene oxide); PA6—polyamide 6; Barrer—10^−10^ cm^3^(STP) cm/(cm^2^ s cmHg); PAMAM—poly(amidoamine) dendrimer; PEGDMA—poly(ethylene glycol) dimethacrylate; GMA—glycidyl methacrylate; PES—polyethersulfone; POP—porous organic polymers; CNC—crystalline nanocellulose; His-NPs—Zinc-coordinated self-assembled histidine-based bolaamphiphile nanoparticles.

**Table 2:** Liquid membranes with carbonic anhydrase for CO_2_ separation.

Application	Membrane Configuration	Enzyme, Concentration	Performance	Year, 1st Author, Ref
CO_2_ separation from N_2_	Microporous PP HFCLM mat with heat exchanger type design (mutually orthogonal fiber orientation)	3 mg/mL CA in 1.0 M NaHCO_3_	At 10% CO_2_ CO_2_ permeance: 90 GPU CO_2_/N_2_ selectivity: 234	2006 Bao [78]
CO_2_ separation from air	Microporous PP HFCLM bundle with feed and sweep fibers intimately commingled	10 mg/L CA in poly(acrylic acid-co-acrylamide) hydrogel	Able to reduce CO_2_ from 0.52% to 0.09%	2008 Cheng [79]
CO_2_ separation from air	Microporous PVDF HFCLM bundle with feed and sweep fibers intimately commingled	121.8 W-A U/L CA displayed on the surface of *E. coli* suspended in water	40% increase in CO_2_ removal rate, 2 times more stable than free CA	2011 Fan [102]
CO_2_ separation from N_2_	SLM with enzyme solution impregnating hydrophilic PVDF membrane; hybrid nylon-silica CLM sandwiched between two hydrophobic PVDF membranes	0.2 mg/mL CA in 1 M NaHCO_3_ pH~8	CO_2_ permeance: 108 GPU, silica xerogel provides additional catalytic benefit	2011 Favre [103]
CO_2_ separation from N_2_	SILM with porous hydrophobic PVDF membrane	0.01 wt% CA in hydrophobic [C_4_MIM][Tf_2_N] ionic liquid or PEG 300	Max CO_2_/N_2_ selectivity: 48; enzyme enhancement is more profound at higher water content	2012 Neves [104]
CO_2_ separation from N_2_, H_2_, CH_4_	SILM with hydrophobic PVDF microfiltration membrane	5 mg/mL in hydrophobic [C_4_MIM][Tf_2_N] ionic liquid	Selectivity: CO_2_/N_2_:30.3 CO_2_/CH_4_:19.9 CO_2_/H_2_:11.2	2016 Bednar [80]
CO_2_ separation from N_2_	SLM with porous hydrophilic cellulose acetate membrane reinforced by pectin	2 mg CA/mL in Tris buffer (20Mm, pH 8.3)	CO_2_ permeability: 93 Barrer CO_2_ permeance: 0.75 GPU (120 μm thickness) CO_2_/N_2_ selectivity: 54	2018 Nemestóthy [82]
CO_2_ separation from N_2_	ILM within 8 nm hydrophilic silica mesopores and thickness of 18 nm	2 CA per nanopore; effective conc. of 100 mg CA mL^–1^	CO_2_ permeance: 2600 GPU Selectivity: CO_2_/N_2_:788; CO_2_/H_2_:1500	2018 Fu [105]
CO_2_ separation from N_2_	SLM with DES filling hydrophilic PTFE microfiltration membrane	0.5 mg CA/g DES (choline chloride and levulinic acid)	CO_2_ permeability: 78 Barrer CO_2_/N_2_ selectivity: 32 Adding CA failed to enhance selectivity	2021 de Castro [81]
CO_2_ separation from N_2_ and CH_4_	SLM with DES filling hydrophilic PTFE microfiltration membrane	0.1 mg CA/mL DES (choline chloride and urea)	CO_2_ permeability: 140 Barrer (w/CA) Selectivity: CO_2_/N_2_: below RUB CO_2_/CH_4_: above (w/o CA) and on (w/CA) RUB	2021 Craveiro [106]
CO_2_ separation from CH_4_	SLM with water-in-oil emulsion filling porous hydrophobic PVDF membrane	1 wt% disperse phase (0.5 g CA/L K_2_CO_3_ pH 11, 5% PEG 300) in corn oil with 2 wt% Tween 80	Permeability of CO_2_ increased by ~15% and CO_2_/CH_4_ selectivity increased by 2.9-fold with CA	2022 Mondal [107]
CO_2_ separation from N_2_ and CH_4_	SLM with hydrophilic PTFE microfiltration membrane	0.5 mg CA/g solvent (12.5/14.5/75.0 wt% ChOH/water/glycerol)	CO_2_ permeability: 81 Barrer Selectivity: CO_2_/N_2_: 90.5	2022 Castro [108]

Acronyms: PP—polypropylene; HFCLM—hollow fiber contained liquid membrane; CA—carbonic anhydrase; GPU—gas permeation unit: 10^−6^ cm^3^(STP)/(cm^2^ s cmHg); PVDF—polyvinylidene fluoride; W-A—Wilbur and Anderson; SLM—supported liquid membrane; CLM—contained liquid membrane; SILM—supported ionic liquid membrane; [C_4_MIM][Tf_2_N]—1-butyl-3-methylimidazolium bis(trifluoromethanesulfonyl)imide; PEG—polyethylene glycol; ILM—immobilized liquid membrane; DES—deep eutectic solvents; RUB—Robeson upper-bound; ChOH—choline hydroxide.

**Table 3:** Gas liquid membrane contactor with carbonic anhydrase for CO_2_ absorption and desorption.

Application	Membrane Configuration	Enzyme, Concentration	Performance	Year, 1st Author, Ref
Artificial lungs CO_2_ desorption	PMP HFGLMC	Immobilized CA up to 88% theoretical monolayer coverage, 0.3 U esterase activity	Rates of CO_2_ exchange from buffer increased by 75% with immobilized CA	2007 Kaar [13]
Artificial lungs CO_2_ desorption	PMP and PP HFGLMC	Immobilized CA 0.99–8.8 U esterase activity	CO_2_ removal rate increased by 115% and 37% from buffer and from blood, respectively	2012–2015 Arazawa [120,121,122,123]
CO_2_ absorption	PP flat sheet membrane with LbL polyelectrolytes PEI/PSS/PAH/MSNP	440 μg CA cm^−2^ per layer tested up to 3 layers	CO_2_ hydration rate of 19 ± 4 μmol cm^−2^ min^−1^ per layer tested up to 3 layers using CO_2_-saturated buffer	2015 Yong [88]
CO_2_ absorption	Hydrophobic PVDF flat sheet membrane with TiO_2_ coating	700 μg CA cm^−2^	CO_2_ hydration rate of 140 μmol cm^−2^ min^−1^ nominal membrane area using CO_2_-saturated buffer	2015 Hou [127]
CO_2_ absorption	PVDF flat sheet Janus membrane with fluorosilane-treated superhydrophobic and CNT-coated hydrophilic sides	165 ± 22 μg CA cm^−2^	CO_2_ hydration rate of 1.32 μmol cm^−2^ min^−1^ from 100% CO_2_ gas to pure water	2015 Hou [128]
CO_2_ absorption	PP- or TiO_2_-coated superhydrophobic PP HFGLMC	200 μg immobilized CA (on TiO_2_ NP)/mL suspended in absorption buffer	CO_2_ hydration rate of 0.96 μmol cm^−2^ min^−1^ from 20% CO_2_ gas mixture to buffer	2016 Hou [129]
CO_2_ absorption	GLMC with a tubular porous glass membrane with hydrophobic coating on the outer skin	10 mM enzyme mimic in 0.5 M K_2_CO_3_	10.8 μmol cm^−2^ min^−1^ from 10% CO_2_ gas mixture to 1 M NaOH; 10-fold increase in rate constant using 10 mM enzyme mimic in 0.5 M K_2_CO_3_	2016 Saeed [130]
CO_2_ absorption	HFGLMC with porous PP or non-porous Psf with PDMS coating; LbL polyelectrolytes PEI/PSS/PAH for enzyme adsorption	Three tri-layers (PSS/PAH/CA) and about 0.15 mg CA in HFGLMC.	3-fold improvement in CO_2_ absorption rate in 30 wt% K_2_CO_3_ with immobilized CA which retained > 80% activity after exposure to common contaminant from flue gas but did not tolerate high pH combined with high temperature	2016–2017 Yong [131,132]
CO_2_ absorption	HFGLMC based on hydrophobic porous PP (bulk and pores) and a dense hydrophilic PVDF layer and PIL coating on flue gas side	1 wt% CA concentrate dissolved in 30% MDEA or MEA.	2.2- and 1.7-fold enzyme enhancement in 30% MDEA with (CO_2_ flux 0.41 μmol cm^−2^ min^−1^ from 15% CO_2_) or without PIL coating, respectively; negative effect for adding CA in 30% MEA	2017 Kim [89]
CO_2_ absorption from CO_2_/Xe mixture	Dense flat sheet PDMS GLMC separating gas and liquid phase microfluidic channels with alveolar design	0.1 mg CA/g of CP ionic liquid with water activity of 0.753	Enzyme has no effect on Xe transport but has 1.9-fold enhancement for CO_2_ absorption	2018 Malankowska [133]
CO_2_ absorption	PVDF HFGLMC with co-deposited PDA/PEI for enzyme immobilization	498 U esterase activity per m^2^ membrane	15 μmol cm^−2^ min^−1^, 150% higher than non-biocatalytic membrane	2019 Xu [134]
CO_2_ absorption	Electrospun PSMA nanofiber membrane as enzyme carrier and gas–liquid contacting surface positioned by flotation device	10 mg CA/mg nanofiber membrane	CO_2_ hydration rate 8.9 μmol cm^−2^ min^−1^ from 100% CO_2_ gas	2020 Kim [135]
CO_2_ absorption	MOF grown on Al_2_O_3_ membrane filter for enzyme adsorption	0.1 mg CA/membrane or 75 μg CA/cm^2^ nominal area	CO_2_ hydration rate 108 μmol cm^−2^ min^−1^ from 5% CO_2_ gas into water	2021 Liu [136]
CO_2_ absorption	Flat sheet PP GLMC with co-deposited PEI/PDA for enzyme immobilization	94.3 µg CA/cm^2^	CO_2_ hydration rate 1.74 μmol cm^−2^ min^−1^ from 15% CO_2_ into 100 mM Tris buffer	2021 Rasouli [137]
CO_2_ absorption	Biocatalytic Flat sheet PP GLMC and MNP both were co-deposited with PEI/PDA and used for enzyme immobilization	6.49–65.44 mg CA/L_reactor_	CO_2_ hydration rate 1.7 μmol cm^−2^ min^−1^ from 15% CO_2_ into 100 mM Tris buffer	2022 Rasouli [90]

Acronyms: PMP—poly(methyl pentene); HFGLMC—hollow fiber gas liquid membrane contactor; CA—carbonic anhydrase; PP—polypropylene; LbL—layer-by-layer assembly; PEI—polyethylenimine; PSS—poly(styrene sulfonate); PAH—poly(allylamine hydrochloride); MSNP—mesoporous silica nanoparticle; PVDF—polyvinylidene fluoride; CNT—carbon nanotube; NP—nanoparticle; GLMC—gas liquid membrane contactor; PSf—polysulfone; PIL—poly(ionic liquids); MDEA—methyldiethanolamine; MEA—monoethanolamine; CP—cholinium propionate; PDA—polydopamine; PSMA—poly(styrene-co-maleic anhydride); Al_2_O_3_—aluminum oxide; MNP—magnetic nanoparticle.

**Table 4:** CO_2_ conversion to chemicals with oxidoreductase.

Application	Membrane Configuration	Enzyme, Concentration	Cofactor Regeneration Electron Transfer System	Year, 1st Author, Ref
CO_2_ conversion to formic acid	Ceramic tubular membrane as UV-light blocker	FDH; DAH	UV > TiO_2_ (EtOH as hole quencher) > MV > DAH > NADH > FDH	2005 Kurayama [92]
CO_2_ conversion to methanol	Flat sheet polymeric membranes with immobilized enzymes by direct membrane fouling	FDH; FaldDH; ADH	NADH > FDH; FaldDH; ADH	2015 Luo [37]
CO_2_ conversion to formic acid	Hydrophobic HFM as gas distributor and PAA-grafted PE HFM as enzyme carrier	FDH	NADH > FDH	2016 Wang [95]
Formaldehyde conversion to methanol	Hydrophilic flat sheet macroporous (200 nm) SiC membrane pretreated with NaOH and surface functionalized with PEI or APTES as enzyme carrier	ADH	NADH > ADH	2018 Zeuner [140]
CO_2_ conversion to formic acid	PAA-grafted PE HFM modified by PEI through electrostatic interaction as CO_2_-philic surface	FDH	NADH > FDH	2018 Wang [141]
CO_2_ conversion to formic acid	Electrospun PS nanofiber membrane surface modified by acid treatment, APTES, and GA activation as enzyme carrier	FDH	Cu foam electrode > NADH > FDH	2018 Barin [142]
CO_2_ conversion to methanol	PVDF porous membrane functionalized by dead-end filtration of MOFs containing enzymes and cofactor	FDH; FaldDH; ADH; GDH	L-glutamate > GDH > NADH > FDH; FaldDH; ADH	2019 Zhu [143]
CO_2_ conversion to formic acid	Porous HFM used as both gas distributor and enzyme carrier	FDH	UV > TiO_2_ (EDTA as hole quencher) > [Cp⁎Rh(bpy) (H2O)]^2+^ > NADH > FDH	2020 Gu [144]
CO_2_ conversion to formic acid	PAA-grafted PE HFM modified by PEI compared with PEI/PDA co-deposited SiO_2_ microsphere as enzyme carriers	FDH	NADH > FDH	2021 Guo [145]
CO_2_ absorption and conversion to formic acid	PP or ceramic GLMC modified by PEI/PDA and in situ grown MOFs encapsulating enzymes	CA; FDH	NADH > FDH	2021 Chai [146]
CO_2_ conversion to formic acid	Ultrafiltration membrane with hydrophobic PP support layer and hydrophilic regenerated cellulose skin layer for enzyme immobilization	FDH	UV > MIL-125-Py-Rh (TEOA as hole quencher) > NADH > FDH	2022 Lin [147]
CO_2_ conversion to formic acid	Ultrafiltration membrane with hydrophobic PP support layer and hydrophilic regenerated cellulose skin layer for enzyme immobilization	FDH	UV > Rh_m3_-N-PCN (TEOA as hole quencher) > NADH > FDH	2022 Zhang [96]
CO_2_ conversion to formaldehyde	PE hollow fiber membrane was used as the enzyme-bearing reactor and gas distributor	FDH; FaldDH	UV > TiO_2_ (EDTA or H_2_O as hole quencher) > [Cp⁎Rh(bpy) (H_2_O)]^2+^ > NADH > FDH; FaldDH	2022 Guo [94]

Acronyms: UV—ultraviolet light; FDH—formate dehydrogenase; MV—methyl viologen; DAH—diaphorase; EtOH—ethanol; FaldDH—formaldehyde dehydrogenase; ADH—alcohol dehydrogenase; HFM—hollow fiber membrane; PAA—poly(acrylic acid); PE—polyethylene; SiC—silicon carbide; NaOH—sodium hydroxide; PEI—polyethylenimine; APTES—(3-aminopropyl)triethoxysilane; GA—glutaraldehyde; MOF—metal organic frameworks; GDH—glutamic dehydrogenase; EDTA—ethylenediaminetetraacetic acid; [Cp⁎Rh(bpy) (H_2_O)]^2+^—pentamethylcyclopentadienyl rhodium bipyridine complex; GLMC—gas–liquid membrane contactor; PDA—polydopamine; TEOA—triethanolamine; MIL-125-Py-Rh—a Ti-based MOF with anchored Rh complex; Rh_m3_-N-PCN—Rhodium complex covalently grafted on amine functionalized polymeric carbon nitride.

## Data Availability

Not applicable.
